# Children’s exposure to television advertising of unhealthy foods and beverages across four countries of WHO European Region

**DOI:** 10.1017/S1368980023000423

**Published:** 2023-12

**Authors:** Anna V Kontsevaya, Asiia E Imaeva, Yulia A Balanova, João J Breda, Kremlin Wickramasinghe, Jo Martin Jewell, Shynar Abdrakhmanova, Andrew G Polupanov, Tulay Bagci Bosi, Toker Ergüder, Oksana M Drapkina, Emma J Boyland

**Affiliations:** 1 National Medical Research Center for Therapy and Preventive Medicine of the Ministry of Healthcare of the Russian Federation, Moscow, Russia; 2 National Medical Research Center for Therapy and Preventive Medicine of the Ministry of Healthcare of the Russian Federation, Department of Epidemiology of Chronic Non-Communicable Diseases, Moscow, Russia; 3 World Health Organization, Regional Office for Europe, Moscow, Russia; 4 World Health Organization, Regional Office for Europe, Copenhagen, Denmark; 5 National Center of Public Health under the Ministry of Health of the Republic of Kazakhstan, Nur-Sultan, Kazakhstan; 6 National Center for Cardiology and Therapy named after academician Mirsaid Mirrakhimov under the Ministry of Health of the Kyrgyz Republic, Bishkek, Kyrgyzstan; 7 Hacettepe University, Department of Public Health, School of Medicine, Ankara, Turkey; 8 World Health Organization, Country Office in Turkey, Ankara, Turkey; 9 Department of Psychology, University of Liverpool, Liverpool, UK

**Keywords:** Food and beverage marketing, advertising, children, adolescents, television

## Abstract

**Objective:**

To compare the frequency and healthfulness of foods being advertised to children and adolescents in four countries of WHO European region.

**Design:**

Cross-sectional quantitative study, guided by an adapted version of the WHO protocol. All recorded food advertisements were categorised by categories and as either ‘permitted’ or ‘not permitted’ for advertising to children in accordance with WHO Regional Office for Europe Nutrient Profile Model.

**Settings:**

Four countries: Russia, Turkey, Kazakhstan and Kyrgyzstan

**Participants:**

TV channels most popular among children and adolescents

**Results:**

Analysis included 70 d of TV broadcasting for all channels, during which time there were 28 399 advertisements. The mean number of advertisements per hour varied from eleven in Turkey and Kazakhstan to eight and two in Russia and Kyrgyzstan. In all countries, the majority of the food and beverages advertised should not be permitted for advertising to children according to the WHO Nutrient Profile Model. The mean number of non-permitted food and beverage advertisements per hour was high in Turkey and Kazakhstan (8·8 and 8·5 ads) compared with Russia (5·1) and Kyrgyzstan (1·9). Turkey was the only country where nutritional information was fully available, and no values were missing that prevented coding for some product categories.

**Conclusions:**

Results revealed that children and adolescents in four countries are exposed to a considerable volume of food and beverage advertisements, including sugary products on broadcast television. As such, policymakers should consider protecting youth by developing regulations to restrict these marketing activities within media popular with children.

The prevalence of childhood obesity has risen substantially in recent decades, making it a serious public health problem. The determinants of childhood obesity are complex and include individual, family and environmental factors^(1)^. One of the factors that has been demonstrated to have an impact on children’s eating behaviour and body weight is children’s exposure to marketing of foods and drinks high in saturated fat, trans fatty acids, free sugars and/or salt (‘HFSS’)^(2)^. Numerous studies have demonstrated that television-based food and beverage marketing directed at children predominantly promotes HFSS foods and drinks. Sugary breakfast cereals, soft drinks, confectionery, snack foods, ready meals and fast foods are the advertised products most often targeted at or seen by children around the world^(3)^. Experimental evidence shows that exposure to HFSS foods and drinks advertising results in a higher intake of energy-dense, sweet and salty foods among those exposed^(4,5)^, particularly in children with overweight and obesity^(6)^, and that this effect meets epidemiological criteria for a causal relationship^(7)^.

Child-oriented food and beverage television advertising also influence children’s preferences and food requests and has been associated with the increased pestering of parents to purchase advertised products, otherwise known as ‘pester power’^(8)^. Adolescents and young adults, in particular, are autonomous in their spending habits and are a particularly valuable target for fast food marketing due to their independent spending power^(9)^. In addition to these known behavioural effects, there is also evidence that food marketing exposure impacts dietary norms, population-level shifts in food and drink category preferences and the cultural values underpinning food behaviours^(10)^.

Based on this evidence, in May 2010, Member States of the WHO endorsed Resolution WHO 63·14, calling for limits on the marketing of food and non-alcoholic beverage products to children. Subsequently, the WHO released the Set of Recommendations to guide efforts by Member States in designing new and/or strengthening existing policies on food marketing communications^(11)^. The WHO has explicitly called on Member States to introduce comprehensive restrictions on marketing of HFSS foods and drinks to children in all media, including television. Governments in a number of European countries have introduced regulations to restrict the advertisement of HFSS foods and drinks on TV (UK, Denmark, Norway, Sweden, France, Slovenia, Turkey, Latvia, Lithuania, Portugal)^(12–16)^; however, the effectiveness of these policies has often been limited due to weak policy designs as well as the migration of advertising to less regulated platforms^(17)^. Many other countries have yet to introduce any advertising restrictions^(12)^; this policy inaction may reflect a lack of specific data on food advertising in these countries.

To be effective, policies should be evidence-based and respond to specific challenges identified; therefore, in states where food marketing restrictions do not currently exist, a first essential step in the policy development process is monitoring the current advertising landscape to build the case for action^(11,12)^. As an important tool for implementing restrictions on the marketing of foods to children, a nutrient profiling tool is recommended. This tool makes it possible to differentiate between foods and non-alcoholic beverages (hereafter ‘foods’) that are more likely to be part of a healthy diet from those that are less likely (in particular, those foods that may contribute to excess energy intake, saturated fats, trans fats, sugar or salt)^(18)^. The WHO European Nutrient Profile Model (WHO-ENPM) for marketing food to children is used by many researchers^(18,19)^.

Existing studies describing the extent and nature of television food advertising are typically based in Australia, Western Europe and North America ^(3)^.

There is very limited data on the food advertising children, and adolescents are exposed to on television in the Commonwealth independent states (CIS) countries and Eastern Europe. However, a recent paper from the authors of the current study demonstrated the substantial exposure of Russian children and adolescents to HFSS foods and drinks advertising on the five TV channels popular with these audiences^(20)^. A recent report from Turkey also demonstrated high exposure to HFSS foods and drinks marketing ^(21)^ and, to the authors’ knowledge, is the only Middle East country to have published data on this issue.

CIS countries in the framework of the Eurasian economic union have common legislation in some aspects, so there is an opportunity to explore the possibility of introducing regional legislation aimed at restricting HFSS foods and drinks marketing.

Poor nutrition, characterised by high salt, low fruit and vegetable intake, is a common problem in the CIS ^(22,23)^. The WHO HBSC survey demonstrated that low percentage of Russian and Turkish adolescents aged 11, 13 and 15 years old consume fruit and vegetables daily and over 20–30 % consume sweets every day ^(24)^. In WHO European Childhood Obesity Surveillance Initiative (COSI) study with 6–9-year-old children, results were more variable, but still only half of the children consumed fruit and vegetables daily in Russia and less than 20 % in Kyrgyzstan ^(25)^. In accordance with these sub-optimal dietary intakes, growing rates of overweight and obesity, including childhood obesity, have been demonstrated as a substantial problem in many countries in recent years, especially in Russian, Eastern Europe and Central Asia countries ^(26–28)^. While the overall rate of increase in children’s BMI seems to have plateaued (at a high level) in high-income countries since 2000, rates continue to increase in low- and middle-income countries^(29)^.

Therefore, the primary aim of the current study was to compare the frequency and healthfulness of foods being advertised to children and adolescents in four countries of WHO European region (CIS and Middle Eastern countries) for the purposes of informing the development of future policies aimed at restricting its impact on the eating behaviours and health of young people.

## Methods

Four countries (Russia, Turkey, Kazakhstan, and Kyrgyzstan) contributed data on television food advertising that had been collected using an adapted version of the WHO protocol ‘Monitoring food and beverage marketing to children via television and the Internet’ ^(30)^. The Russian and Turkish studies were performed in spring 2017 and the Kazak and Kyrgyz studies in spring 2018.

Training and support in the use of the protocol and in the coding procedure were provided by the authors of the protocol – WHO (JMJ, JB) and academic (EB) experts for Russia and Turkey and by Russian experts (JB and AI) for Kazakhstan and Kyrgyzstan.

### Sampling

In each country, television channels most popular among children and adolescents were identified for monitoring so that the data would best reflect the likely exposure of this demographic to HFSS foods and drinks advertising.

In Russia, publicly available television viewing data were consulted^(31)^ to inform this decision, and the study focussed on federal channels as they are broadcast throughout the country. The following channels were selected: «Карусель»/«Karusel», «Disney», (both child-oriented channels) «СТС»/STS, «ТНТ»/TNT, and «Пятница»/«Piatnitsa» (adolescent-oriented channels).

In Turkey, the five commercial TV channels (A HABER, ATV, KANAL D, SHOW TV, STAR) most popular among young people under 16 years were selected according to the viewing ratings for TV channels as of 1 April 2017 (CanliTV, 2017) were selected. National channels were chosen as these are the only ones subject to regulation by the national broadcast authority ^(32)^.

In Kazakhstan, television broadcasting is done through national and regional distribution channels, cable and satellite channels. There was one national channel for children ‘Balapan’ with the Kazakh language of broadcasting. The other five channels (1 Channel Eurasia, NTK, 31 Channel, Astana TV, Qazaqstan) were selected taking into account national data providing relevant information on the following criteria: popularity, accessibility, national broadcasting and coverage of a children’s audience (children under 16) ^(33)^.

All television channels in Kyrgyzstan can be divided into two groups: federal channels, available throughout the country and included in the basic television package and regional and cable channels. The monitored channels were selected based on the results of media research, social surveys and official statistics, taking into account their popularity and data on the size of the child and adolescent audience. The following six national television channels were chosen for the current study: ‘Balastan’, ‘312 cinema’, ‘KTRK’, ‘KTRK Muzyka’, ‘KTRK Sport’ and ‘Tumar’.

### Data collection

Data collection was performed in 2017–2018. In all countries, TV broadcasts on each channel were recorded by the research team for weekdays and weekend days, from 6 am to 10 pm (16 h). The recording days were chosen by random sampling and excluded periods of national holidays. The total number of recording days varied by country. In Russia, the sample comprised 20 d of recording (10 weekdays and 10 weekend days) between March and May 2017. In Turkey, it included only 2 d (1 weekday and 1 weekend day) during the first week of April 2017 (on 6th and 9th of April). In Kazakhstan and Kyrgyzstan, samples included 24 d of recording (12 weekdays and 12 weekend days) between March and May 2018.

The full sets of recordings were coded for food marketing to children.

Adaptations to the WHO protocol ‘Monitoring food and beverage marketing to children via television and the Internet’ ^(30)^ to meet the specifics of the Russian Federation included incorporating social marketing, sausage factories, dairy production, infant formula and advertising the product range without highlighting any particular dish (i.e. promoting the assortment that the company offered in its product portfolio), as advertisement types, and adding some culturally relevant categories to the coding system for food and beverages advertisements (specifically non-alcoholic beer and tea were included in the category beverages).

As a result, the list of advertisement types for coding has been extended (online Supplementary Table S1). The study protocols for Kyrgyzstan and Kazakhstan were modified, according to the countryʼs specificities, by adding such categories as social advertising, sports goods and entertainment via SMS format.

All recordings were viewed and screened for spot advertisements (those shown between and during programs). Other forms of marketing such as product placement and program sponsorship were not included. All advertisements were coded into one of twenty-seven different types (online Supplementary Table S1) by two researchers. In cases where there was more than one food item in the advertisement, the one that was presented first was coded.

In order to ensure the reliability of coding across the two researchers, both researchers initially coded 1 d of data for one channel, according to the predefined criteria set out in the research protocol. After that, project managers checked the coding to remove any inconsistencies, the results were compared, discrepancies discussed and agreement was reached for all instances of disagreement. Then the research assistants coded these advertisements again.

Food and beverages advertisements (defined as those featuring a food item for sale, such as from a food retailer or fast food restaurant) were then additionally coded in accordance with seventeen food and beverages categories described in the WHO Regional Office for Europe Nutrient Profile Model (NPM)^(34)^.

### Nutritional analysis

The WHO NPM was used to classify foods and beverages as permitted or not permitted to be marketed to children. The model does this using first a category-level classification and then, for some categories, there are additional nutrient thresholds that must be met for marketing to be permitted. For example, a product categorised as ‘chocolate and sugar confectionery’ is not permitted to be marketed to children regardless of the nutrient content, but within the category ‘breakfast cereals’ a product may be permitted to be marketed to children if the total fat, total sugar and salt levels per 100 g of product are below the stated thresholds. Therefore, for some products, it was necessary to obtain the nutrition information, and where possible, this information was sourced from product packaging (accessed online or at point of sale in retail stores) ^(34)^. In the Russian Federation, as well as in two other CIS countries, nutritional information on product packaging on the amount of salt, added sugar or trans-fat is not mandatory and so often is not provided. Therefore, in some cases, it was not possible to make a judgement as to whether the marketing of that product would be permitted according to WHO NPM.

### Data analysis

Coding for all variables was entered directly into Microsoft Excel while viewing the TV recordings. All final datasets were then provided to the Russian team for the combined analysis. Statistical analysis was performed using SPSS version 21·0 software for Windows (SPSS Inc, Chicago, IL).

Analyses were conducted to address the following research questions:

1. What is the quantity of food and beverage advertising on TV popular with children and adolescents in these four countries, and does this differ by country?

2. What WHO Europe NPM food and beverage categories are promoted the most, and does this differ between countries?

3. What proportion of food and beverage ads on TV stations popular with children and adolescents are classified as permitted and non-permitted according to the WHO Europe NPM, and does this differ by country?

4. Does the hourly rate of ‘not permitted’ food and beverage advertising differ by country?

## Results

### Sample description

Four research groups from four countries contributed data for this research. The final compiled dataset spanned countries from the Russian Federation, Turkey, Kazakhstan and Kyrgyzstan (Table 1). During the analysis, it was observed that three channels in Kyrgyzstan had no advertisements, while one channel in Kazakhstan broadcast only 100 advertisements over 32 h.

**Table 1:** Recorded TV sample description by country

	Time period of data collection	Number of channels monitored	Weekdays/Weekend days per channel	Total number of d/h	Total advertisements recorded
Russian Federation	18·03·17–31·05·17	5	2/2	20/320	11 638
Turkey	06·04·17–09·04·17	5	1/1	10/160	3962
Kazakhstan	29·03·18–20·05·18	6	2/2	24/384	10 641
Kyrgyzstan	25·03·18 –27·05·18	6	2/2	24/384	2158

### Overall volume of food and beverage advertising by country

**Fig. 1:**
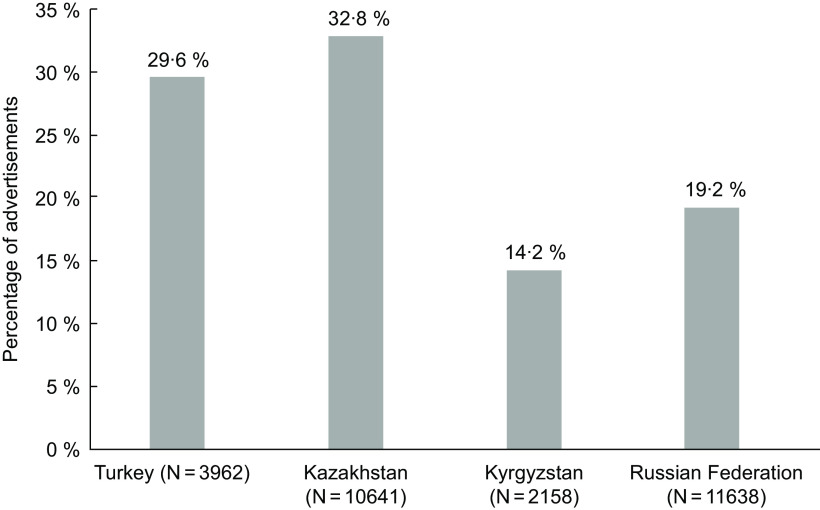
Proportion of advertisements that were for foods or beverages in the four countries studied.

Analysis included 78 d of TV broadcasting for all channels, during which time there were 28 399 advertisements. The mean number of advertisements per day varied from 584.0 in Russia to 89.9 in Kyrgyzstan. Across countries, 14–32 % of advertisements were for food or beverage products, with the greatest proportion in Kazakhstan (32·8 %) and the lowest in Kyrgyzstan (14·2 %) (Fig. 1). The rate of food advertising per day was highest in Turkey and Kazakhstan (141·2 and 145·5, respectively) but substantially lower in Kyrgyzstan (12·8).

### Nutritional quality of foods and beverages advertised by country

There was some diversity in the top five most commonly advertised food and beverage categories by country, with some similarities between Kazakhstan and Kyrgyzstan (Table 2). The most frequently advertised food and beverage categories in Turkey were the following: chocolate and sugar confectionery (20·7 % of food ads), edible ices, including ice cream (18·8 %), mineral and sweetened beverages, including cola, lemonade, mineral and/or flavoured waters (14·1 %), savoury snacks (7·6 %), and cakes, sweet biscuits and pastries (6·9 %). In Kyrgyzstan and Kazakhstan, the most frequently advertised category was mineral and sweetened beverages, including cola, lemonade, mineral and/or flavoured waters (21·9 % and 49·7 %, respectively). Chocolate and sugar confectionery was the second most commonly advertised category in Kazakhstan (17·4 %) and the fourth in Kyrgyzstan (7·2 %). The fifth category was also similar – milk drinks, including milks and sweetened milks (6·4 % and 7·2 %). In Russia, the most frequently advertised product category was yogurts, sour milk and cream (15·5 %), which was also in the third position in Kazakhstan (10·6 %) but was not in the top five categories in the other two countries. In Russia, the next most frequently advertised product category was chocolate and sugar confectionery (12·3 %) which was rather frequent in all countries in this analysis. Mineral and sweetened beverages, including cola, lemonade, mineral and/or flavoured waters (another commonly advertised product in all countries) were third in Russia (10·9 %). Detailed information for each channel included in the analysis is presented in the supplementary materials (online Supplementary Table S2).

**Table 2:** Top five food and beverage categories advertised by country, using WHO regional office for Europe nutrient profiling model

	1st	2rd	3rd	4th	5th
Turkey (*n* 1273)	20·7 % (сhocolate, sugar confectionery, etc.)	18·8 % (edible ices, including ice cream, etc.)	14·1 % mineral and sweetened beverages, including cola, lemonade, mineral and/or flavoured waters, etc.)	7·6 % (savoury snacks, etc.)	6·9 % (cakes, sweet biscuits, pastries, etc.)
Kazakhstan (*n* 3494)	21·9 % mineral and sweetened beverages, including cola, lemonade, mineral and/or flavoured waters, etc.)	17·4 % (сhocolate, sugar confectionery, etc.)	10·6 % (yogurts, sour milk, cream, etc.)	7·7 % (tea, coffee)	6·4 % (milk drinks (including milks and sweetened milks, etc.)
Kyrgyzstan (*n* 153)	49·7 % mineral and sweetened beverages, including cola, lemonade, mineral and/or flavoured waters, etc.)	18·3 % (juices)	17·0 % (savoury snacks, etc.)	7·2 % (сhocolate, sugar confectionery, etc.)	7·2 % (milk drinks (including milks and sweetened milks, etc.)
Russian Federation (*n* 2248)	15·0 % (yogurts, sour milk, cream, etc.)	12·3 % (сhocolate, sugar confectionery, etc.)	10·9 % mineral and sweetened beverages, including cola, lemonade, mineral and/or flavoured waters, etc.)	10·0 % (ready-made and convenience foods, composite dishes, etc.)	9·8 % (tea, coffee)

**Fig. 2:**
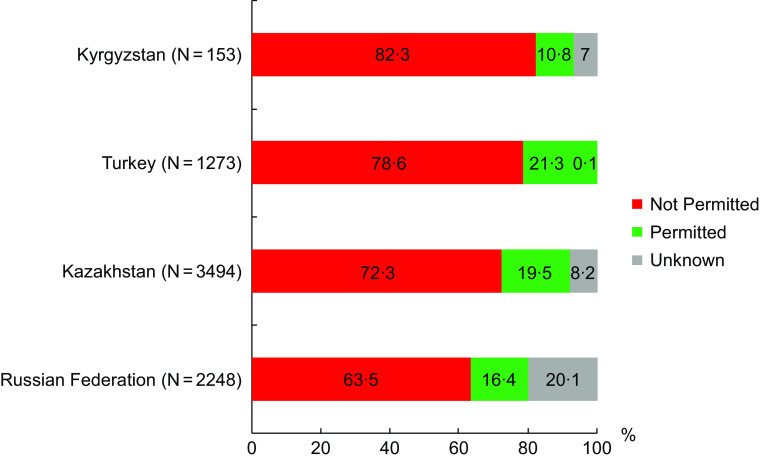
The proportion of food advertisements per country classified as permitted, not permitted or unknown for advertising to children according to the WHO Nutrient Profile Model for Europe.

In all countries, the majority of the advertised food and beverages should not be permitted for advertising to children, according to the WHO NPM (Fig. 2). Turkey was the only country where nutritional information was fully available, and no values were missing that prevented coding for some product categories. In other countries, the percentage of advertisements that could not be classified according to the WHO NPM varied from 20 % to 7 %.

The mean number of non-permitted food and beverage advertisements per hour was high in Turkey and Kazakhstan (8·8 and 8·5 ads) compared with Russia (5·1) and Kyrgyzstan (1·9). The mean number of ads that could not be classified using the WHO NPM for three CIS countries were 2·1, 1·1 and 2·3 ads per hour in Kazakhstan, Kyrgyzstan and Russian Federation, respectively) (Table 3). Detailed information for each channel included in the analysis is presented in the supplementary materials (online Supplementary Table S3 and S4).

**Table 3:** Average frequency of food and beverage advertising, applying the WHO regional office for Europe nutrient profiling model per hour

Countries/channels	Not permitted		Permitted		Unknown		All food	
	Mean	sd	Mean	sd	Mean	sd	Mean	sd
Turkey	8·8	4·7	2·9	1·8	–		11·1	5·8
Kazakhstan	8·5	5·9	2·9	2·0	2·1	1·6	11·4	8·2
Kyrgyzstan	1·9	1·1	1·0	0·0	1·1	0·3	2·1	1·3
Russian Federation	5·1	7·4	2·2	4·2	2·3	1·9	7·9	11·8

## Discussion

This study is the first to report comparative data on the frequency and healthfulness of foods being advertised to which children and adolescents in four countries of the WHO European regions (including CIS countries) are likely to be exposed, and therefore may be used to inform policy development across this region.

The proportion of food advertisements ranged from 14·2 to 32·8 %. This is consistent with the proportion of food advertising found in previous studies in Germany (18·5 %)^(35)^, Australia and China (both 25 %) ^(36,37)^ and slightly higher than in the UK (12·8 %), although it should be noted that the statutory regulation of food marketing was partly implemented in the UK when this study was conducted^(38)^.

The study shows problems with the classification of foods due to poor information about food composition in CIS countries, as 7–20 % of advertisements could not be classified according to the WHO Nutrient Profile Model for Europe. It is a clear sign to improve the food labelling in these countries, as the lack of data is a serious barrier to a healthy food choice ^(39)^.

The highest number of not permitted food advertisements per hour were in Turkey and Kazakhstan (8·8 and 8·5, respectively). However, in Turkey, all advertisements could be classified into categories, whereas this was not the case for Kazakhstan or the other two countries.

The lowest rate of exposure for not permitted food advertisement was in Kyrgyzstan (1·9 per hour), but this reflects a much lower frequency of TV advertising overall compared with other countries in this analysis rather than a more healthy profile of advertising (indeed, the proportion of not permitted food advertisements was highest here – 82·3 %). The average frequency was higher in the other three countries compared to the published results from 22 countries ^(3)^ and in some recent studies ^(40)^.

The top five most frequently advertised food and beverage categories in four countries included several similar food groups, such as chocolate and confectionery, ready-made food and dishes (including fast food), 78·6 % of which were unhealthy versions that exceeded the WHO NPM threshold criteria for fats, sugar, Na and/or energy. Recent systematic review and meta-analysis demonstrated that food marketing was associated with increased intake, choice, preference and purchase requests in children and adolescents ^(41)^.

Fast food advertisements have previously been associated with an increase in fast food consumption and an increased risk of obesity in children ^(6,42)^ and sugar-sweetened beverages have been shown to be the main source of added sugar in young people’s diets, contributing to poorer lipid profiles that increase risk of negative health outcomes such as CVD and stroke ^(42)^.

Findings from the current study may have important policy implications. Results revealed that children and adolescents in four countries, including three CIS, are likely to have been exposed to a considerable volume of food and beverage advertisements, including sugary products on broadcast television. As such, policymakers should consider protecting youth by developing regulations to restrict these marketing activities within media popular with children. Evaluations from countries with restrictive food marketing policies in place show that it is possible to achieve desirable reductions in exposure and associated behavioural outcomes, such as purchasing of unhealthy products ^(43,44)^. To maximise effectiveness, policymakers should seek to apply regulations to all programming to which children are exposed, not just that which is directed specifically at youth audiences ^(45)^. To address this, some countries such as the UK have proposed so-called ‘watershed’ bans whereby HFSS foods and drinks advertising cannot be shown until after 9 or 10 pm. Modelling studies suggest such policies would be cost-effective and achieve meaningful reductions in childhood obesity, particularly for more deprived children ^(46,47)^.

It is clear that the countries in which restrictive food marketing policies have been implemented are typically also those countries with substantial data on advertising prevalence derived from monitoring studies ^(3)^. In line with the WHO recommendations, which note the importance of monitoring and evaluation mechanisms to underpin the policy cycle, it is crucial that national monitoring systems are established to gather robust data on key indicators (such as exposure and behavioural impacts). No CIS country currently has regulations on food marketing for children. Considering that three CIS countries have common legislation in certain aspects, there is a window of opportunity to introduce new legislation at the level of Eurasian economic union, and this study provides timely data to support initial discussions towards this important public health policy target.

### Limitations

This study did not measure all aspects of food marketing on television, for example, program sponsorship or product placement in children’s movies ^(47)^, nor did it consider other platforms for food marketing (such as sports sponsorship, outdoor advertising) for a more comprehensive assessment of children’s likely exposure. It was possible only to estimate the potential exposure without considering the number of children viewing the advertisement (i.e. the reach of the advertisement). Also we focused on the TV channels most popular with children and adolescents and did not monitor food advertising on the other channels watched by young people (such as those carrying family entertainment shows), and as a result, not all food advertising was evaluated.

## Conclusion

This study adds to the body of literature examining television food advertising directed at children and adolescents by describing the current television food advertising environment in the WHO European region, including three CIS countries. The large volumes of television advertising for HFSS foods and drinks impact children worldwide, including in the four countries that have been analysed in this study. Across all countries, television food and beverage advertisements are predominantly for products that exceed WHO maximum thresholds for saturated fat, Na and/or sugar for foods and beverages that are considered appropriate to be marketed to children. Monitoring data such as those presented in this study can be used as part of evidence-informed policymaking.

## Supplementary material

For supplementary material accompanying this paper visit https://doi.org/10.1017/S1368980023000423. click here to view supplementary material
