# Supplementary material for: Children’s exposure to television advertising of unhealthy foods and beverages across four countries of WHO European Region
Source: Public Health Nutr. 2023 Mar 13;26(Suppl 1):s32–40. doi: 10.1017/S1368980023000423 (PMC10801364; doi:10.1017/S1368980023000423)
Supplement: Supplementary file 1 [file S1368980023000423sup001.docx]

Annex 1

Table S1. List of type of advertisements

| Type of advertisement |
| --- |
| 1 Pharmaceuticals |
| 2. Channel promotions |
| 3. Education |
| 4. Information announcements, sponsored by food companies |
| 5. Infant formula |
| 6. Financial |
| 7. Household cleaners/detergents |
| 8. Toiletries |
| 9. Utilities |
| 10. Social advertisements |
| 11. Clothes/Shoes |
| 12. Food and beverages |
| 13. Household equipment |
| 15. Retailing and mail order |
| 16. Toys |
| 17. Milk processing plant/ meat processing plant |
| 18. Motoring |
| 19. Entertainment |
| 20. Publishing |
| 21. Pet products |
| 22. Travel/Transport/Holidays |
| 23. Public service announcements |
| 24. Food company |
| 25. Сultural and sporting events |
| 26. Sports goods |

Table S2. Top five food and beverage categories advertised by country, using WHO Regional Office for Europe nutrient profiling model (% of food and beverage advertisements for that channel)

| Countries / channels | 1^st^ | 2^rd^ | 3^rd^ | 4^th^ | 5^th^ |
| --- | --- | --- | --- | --- | --- |
| **Turkey**  **(N=1273)** | 20.7% (сhocolate, sugar confectionery etc.) | 18.8% (edible ices, including ice cream etc.) | 14.1% ( mineral and sweetened beverages, including cola, lemonade, mineral and/or flavoured waters etc.) | 7.6% (savoury snacks etc.) | 6.9% (cakes, sweet biscuits, pastries etc.) |
| Channel 1 (N=7) | 85.7% (mineral and sweetened beverages, including cola, lemonade, mineral and/or flavoured waters etc.) | 14.3% (сhocolate, sugar confectionery etc.) | - | - | - |
| Channel 2 (N=298) | 24.2% (сhocolate, sugar confectionery etc.) | 14.4% (edible ices, including ice cream etc.) | 13.1% (mineral and sweetened beverages, including cola, lemonade, mineral and/or flavoured waters etc.) | 7.7% (cakes, sweet biscuits, pastries etc.) | 6.7% (ready-made and convenience foods, composite dishes etc.) |
| Channel 3 (N=279) | 21.5% (edible ices, including ice cream etc.) | 17.9% (сhocolate, sugar confectionery etc.) | 15.4% (mineral and sweetened beverages, including cola, lemonade, mineral and/or flavoured waters etc.) | 11.8% (savoury snacks etc.) | 6.8% (cakes, sweet biscuits, pastries etc.) |
| Channel 4 (N=376) | 21.3% (edible ices, including ice cream etc.) | 17.8% (сhocolate, sugar confectionery etc.) | 13.3% (mineral and sweetened beverages, including cola, lemonade, mineral and/or flavoured waters etc.) | 9.6% (savoury snacks etc.) | 7.4% (cakes, sweet biscuits, pastries etc.) |
| Channel 5 (N=313) | 23.5% (сhocolate, sugar confectionery etc.) | 18.0% (edible ices, including ice cream etc.) | 13.2% (mineral and sweetened beverages, including cola, lemonade, mineral and/or flavoured waters etc.) | 8.0% (ready-made and convenience foods, composite dishes etc.) | 7.4.% (fresh and frozen meat, poultry, fish etc.) |
| **Kazakhstan**  **(N=3494)** | 21.9% (mineral and sweetened beverages, including cola, lemonade, mineral and/or flavoured waters etc.) | 17.4% (сhocolate, sugar confectionery etc.) | 10.6% (yoghurts, sour milk, cream etc.) | 7.7% (tea, coffee) | 6.4% (milk drinks (including milks and sweetened milks) etc.) |
| Balapan  (N=39) | 79.5% (сhocolate, sugar confectionery etc.) | 20.5% (mineral and sweetened beverages, including cola, lemonade, mineral and/or flavoured waters and etc.) | - | - | - |
| NTK (N=731) | 26.6% (сhocolate, sugar confectionery etc.) | 23.4% (mineral and sweetened beverages, including cola, lemonade, mineral and/or flavoured waters etc.) | 8.1% (sauces, dips, dressings etc.) | 8.1% (ready-made and convenience foods, composite dishes etc.) | 7.3 (tea, coffee) |
| Channel 1 Eurasia (N=564) | 26.6% (сhocolate, sugar confectionery etc.) | 21.1% (mineral and sweetened beverages, including cola, lemonade, mineral and/or flavoured waters etc.) | 10.6% (sauces, dips, dressings etc.) | 9.9% (tea, coffee) | 9.0% (yoghurts, sour milk, cream etc.) |
| Channel 31 (N=1206) | 19.8% (mineral and sweetened beverages, including cola, lemonade, mineral and/or flavoured waters etc.) | 12.8% (сhocolate, sugar confectionery etc.) | 11.1% (yoghurts, sour milk, cream etc.) | 8.1% (savoury snacks etc.) | 7.4% (milk drinks (including milks and sweetened milks) etc.) |
| Astana TV (N=671) | 21.9% (mineral and weetened beverages, including cola, lemonade, mineral and/or flavoured waters etc.) | 15.4% (yoghurts, sour milk, cream etc.) | 8.6% (milk drinks (including milks and sweetened milks) etc.) | 8.6% (cheese (including medium-hard, hard and soft cheeses) etc.) | 7.5% (sauces, dips, dressings etc.) |
| Qazaqstan (N=283) | 28.6% (mineral and sweetened beverages, including cola, lemonade, mineral and/or flavoured waters etc.) | 18.0% (yoghurts, sour milk, cream etc.) | 15.2% (tea, coffee) | 12.4% (сhocolate, sugar confectionery etc.) | 6.4% (milk drinks (including milks and sweetened milks) etc.) |
| **Kyrgyzstan (N=153)** | 49.7% (mineral and weetened beverages, including cola, lemonade, mineral and/or flavoured waters etc.) | 18.3% (juices) | 17.0% (savoury snacks etc.) | 7.2% (сhocolate, sugar confectionery etc.) | 7.2% (milk drinks (including milks and sweetened milks) etc.) |
| 312 Kino* | - | - | - | - | - |
| TumarTV * | - | - | - | - | - |
| Balastan* | - | - | - | - | - |
| KTRK (N=38) | 28.9% (mineral and sweetened beverages, including cola, lemonade, mineral and/or flavoured waters etc.) | 26.3% (savoury snacks etc.) | 23.7% (juices) | 13.2% (milk drinks (including milks and sweetened milks) etc.) | 7.9% (сhocolate, sugar confectionery etc.) |
| KTRK Muzyka (N=80) | 41.3% (mineral and sweetened beverages, including cola, lemonade, mineral and/or flavoured waters etc.) | 23.8% (juices) | 20.0% (savoury snacks etc.) | 7.5% (milk drinks (including milks and sweetened milks) etc.) | 7.5% (сhocolate, sugar confectionery etc.) |
| KTRK Sport (N=35) | 91.4% (mineral and sweetened beverages, including cola, lemonade, mineral and/or flavoured waters etc.) | 5.7% (сhocolate, sugar confectionery etc.) | 2.9% (ready-made and convenience foods, composite dishes etc.) | - | - |
| **Russian Federation (N=2248)** | 15.0% (yoghurts, sour milk, cream etc.) | 12.3% (сhocolate, sugar confectionery etc.) | 10.9% (mineral and sweetened beverages, including cola, lemonade, mineral and/or flavoured waters etc.) | 10.0% (ready-made and convenience foods, composite dishes etc.) | 9.8% (tea, coffee) |
| Disney (N=349) | 30.1% (yoghurts, sour milk, cream etc.) | 16.0% (сhocolate, sugar confectionery etc.) | 13.8% (tea, coffee) | 9.7% (mineral and weetened beverages, including cola, lemonade, mineral and/or flavoured waters etc.) | 7.2% (juices) |
| Karusel  (N=151) | 32% (yoghurts, sour milk, cream etc.) | 16.7% (сhocolate, sugar confectionery etc.) | 13.3% (juices) | 11.3% (mineral and sweetened beverages, including cola, lemonade, mineral and/or flavoured waters etc.) | 9.3% (tea, coffee) |
| Pyatnitsa (N=581) | 14.5% (mineral and sweetened beverages, including cola, lemonade, mineral and/or flavoured waters etc.) | 13.1% (сhocolate, sugar confectionery etc.) | 11.4% (yoghurts, sour milk, cream etc.) | 9.1% (processed meat, poultry, fish and similar (including sausage) etc.) | 9.0% (ready-made and convenience foods, composite dishes etc.) |
| STS (N=583) | 15.4% (ready-made and convenience foods, composite dishes etc.) | 12.5% (сhocolate, sugar confectionery etc.) | 11.0% (yoghurts, sour milk, cream etc.) | 11.0% (tea, coffee) | 9.8% (mineral and sweetened beverages, including cola, lemonade, mineral and/or flavoured waters etc.) |
| TNT (N=584) | 15.1% (processed meat, poultry, fish and similar (including sausage) etc.) | 14.2% (ready-made and convenience foods, composite dishes etc.) | 9.9% (tea, coffee) | 9.4% (yoghurts, sour milk, cream etc.) | 9.1% (mineral and sweetened beverages, including cola, lemonade, mineral and/or flavoured waters etc.) |

Note: * - no food and beverage advertisements

Table S3. Average frequency of food and beverage advertising per day, applying the WHO Regional Office for Europe nutrient profiling model

|  | All Food | Permitted | Not Permitted | Unknown |
| --- | --- | --- | --- | --- |
| **Turkey (N=1273)** | 141.2 ± 54.3 | 33.8 ± 11.0 | 111.1 ± 41.2 | - |
| Channel 1 | 3.5 ± 0.0 | - | 3.5 ± 0.0 | - |
| Channel 2 | 149.0 ± 9.9 | 33.0 ± 7.1 | 115.5 ± 17.7 | - |
| Channel 3 | 139.5 ± 7.8 | 20.5 ± 6.4 | 119.0 ± 1.4 | - |
| Channel 4 | 188.0 ± 22.6 | 45.5 ± 10.6 | 142.5 ± 12.0 | - |
| Channel 5 | 155.5 ± 3.5 | 36.0 ± 2.8 | 119.5 ± 0.7 | - |
| **Kazakhstan (N=3494)** | 145.5 ± 98.1 | 34.2 ± 20.0 | 105.2 ± 67.3 | 17.8 ± 14.4 |
| Balapan | 9.8 ± 0.5 | - | 9.8 ± 0.5 | - |
| NTK | 182.5 ± 44.8 | 28.3 ± 8.4 | 149.0 ± 37.4 | 5.3 ± 0.5 |
| 1 channel Eurasia | 141.0 ± 26.8 | 28.8 ± 8.1 | 103.5 ± 22.0 | 11.7 ± 1.2 |
| 31 channel | 301.5 ± 13.4 | 61.3 ± 23.0 | 202.3 ± 22.2 | 38.0 ± 6.1 |
| Astana TV | 167.8 ± 55.6 | 33.3 ± 20.0 | 115.5 ± 25.8 | 19.0 ± 10.9 |
| Qazaqstan | 70.8 ± 29.1 | 19.3 ± 10.8 | 51.3 ± 20.7 | - |
| **Kyrgyzstan (N=153)** | 12.8 ± 6.0 | 1.2 ± 0.4 | 10.8 ± 6.2 | 3.7 ± 2.1 |
| 312 kino* | - | - | - | - |
| TumarTV* | - | - | - | - |
| Balastan* | - | - | - | - |
| KTRK | 9.5 ± 2.5 | 1.0 ± 0.0 | 7.3 ± 3.5 | 2.5 ± 0.7 |
| KTRK Muzyka | 20.0 ± 3.6 | 1.5 ± 0.6 | 18.5 ± 3.5 | - |
| KTRK Sport | 8.8 ± 2.5 | 0.5 ± 0.0 | 6.8 ± 1.0 | 1.5 ± 0.0 |
| **Russian Federation (N=2248)** | 112.4 ± 54.1 | 19.3 ± 12.2 | 69.5 ± 35.7 | 23.6 ± 17.5 |
| Pyatnitsa | 145.3 ± 33.2 | 16.3 ± 3.3 | 86.0 ±18.1 | 43.0 ± 20.9 |
| Disney | 87.3 ± 27.2 | 22.0 ± 6.4 | 52.8 ± 21.6 | 12.5 ± 7.7 |
| Karusel | 37.5 ± 6.6 | 7.8 ± 3.2 | 22.8 ± 6.8 | 7.0 ± 3.4 |
| STS | 145.8 ± 23.0 | 23.5 ± 7.7 | 92.8 ± 15.4 | 29.5 ± 11.8 |
| TNT | 146.0 ± 58.0 | 26.8 ± 22.9 | 93.3 ± 43.2 | 26.0 ± 14.7 |

Note: * - no food and beverage advertisements

Table S4. Average frequency of food and beverage advertising per hour, applying the WHO Regional Office for Europe nutrient profiling model

| Countries/channels | Not Permitted | Permitted | Unclassified | All food |
| --- | --- | --- | --- | --- |
| **Turkey (N=1273)** | 8.8 ± 4.7 | 2.9 ± 1.8 | - | 11.1 ± 5.8 |
| Channel 1 | 1.4 ± 0.55 | - | - | 1.4 ± 0.55 |
| Channel 2 | 8.6 ± 4.9 | 3.1 ± 1.7 | - | 11.0 ± 5.8 |
| Channel 3 | 8.5 ± 4.3 | 2.0 ± 1.1 | - | 10.0 ± 4.5 |
| Channel 4 | 11.4 ± 4.5 | 3.5 ± 2.2 | - | 14.5 ± 6.5 |
| Channel 5 | 8.5 ± 3.9 | 2.9 ± 1.7 | - | 11.1 ± 4.5 |
| **Kazakhstan (N=3494)** | 8.5 ± 5.9 | 2.9 ± 2.0 | 2.1 ± 1.6 | 11.4 ± 8.2 |
| Balapan | 1.3 ± 0.6 | - | - | 1.3 ± 0.6 |
| NTK | 10.8 ± 5.1 | 2.3 ± 1.4 | 1.0 ± 0.0 | 13.0 ± 6.0 |
| 1 channel Eurasia | 7.0 ± 3.1 | 2.4 ± 1.4 | 1.3 ± 0.5 | 9.4 ± 4.3 |
| 31 channel | 13.5 ± 6.6 | 4.2 ± 2.4 | 2.9 ± 1.6 | 20.1 ± 8.2 |
| Astana TV | 10.3 ± 4.8 | 3.3 ± 2.2 | 2.4 ± 1.9 | 13.4 ± 7.7 |
| Qazaqstan | 4.1 ± 3.0 | 2.0 ± 1.3 | - | 5.5 ± 4.0 |
| **Kyrgyzstan (N=153)** | 1.9 ± 1.1 | 1.0 ± 0.0 | 1.1 ± 0.3 | 2.1 ± 1.3 |
| 312 kino* | - | - | - | - |
| TumarTV* | - | - | - | - |
| Balastan* | - | - | - | - |
| KTRK | 2.1 ± 1.1 | 1.0 ± 0.0 | 1.0 ± 0.0 | 2.5 ± 1.4 |
| KTRK Muzyka | 2.1 ± 1.2 | 1.0 ± 0.0 | - | 2.2 ± 1.4 |
| KTRK Sport | 1.4 ± 0.7 | 1.0 ± 0.0 | 1.2 ± 0.4 | 1.6 ± 0.9 |
| **Russian Federation (N=2248)** | 5.1 ± 7.4 | 2.2 ± 4.2 | 2.3 ± 1.9 | 7.9 ± 11.8 |
| Pyatnitsa | 5.3 ± 2.6 | 1.6 ± 0.7 | 3.0 ± 1.7 | 8.9 ± 3.3 |
| Disney | 3.5 ± 2.1 | 2.0 ± 1.1 | 1.6 ± 0.7 | 5.5 ± 2.7 |
| Karusel | 2.0 ± 1.1 | 1.6 ± 0.8 | 1.2 ±0.5 | 2.8 ± 1.9 |
| STS | 5.8 ± 2.2 | 1.9 ± 0.9 | 2.3 ± 1.2 | 9.1 ± 2.9 |
| TNT | 6.3 ± 3.1 | 2.4 ± 1.6 | 2.1 ± 1.2 | 9.4 ± 4.2 |

Note: * - no food and beverage advertisements
